# Development of Perovskite-Type Materials for Thermoelectric Application

**DOI:** 10.3390/ma11060999

**Published:** 2018-06-12

**Authors:** Tingjun Wu, Peng Gao

**Affiliations:** 1 CAS Key Laboratory of Design and Assembly of Functional Nanostructures, and Fujian Key Laboratory of Nanomaterials, Fujian Institute of Research on the Structure of Matter, Chinese Academy of Sciences, Fuzhou 350002, China; twu@fjirsm.ac.cn; 2 Laboratory of Advanced Functional Materials, Xiamen Institute of Rare Earth Materials, Haixi Institute, Chinese Academy of Sciences, Xiamen 361021, China

**Keywords:** perovskite, thermoelectric, generator, cooler

## Abstract

Oxide perovskite materials have a long history of being investigated for thermoelectric applications. Compared to the state-of-the-art tin and lead chalcogenides, these perovskite compounds have advantages of low toxicity, eco-friendliness, and high elemental abundance. However, because of low electrical conductivity and high thermal conductivity, the total thermoelectric performance of oxide perovskites is relatively poor. Variety of methods were used to enhance the TE properties of oxide perovskite materials, such as doping, inducing oxygen vacancy, embedding crystal imperfection, and so on. Recently, hybrid perovskite materials started to draw attention for thermoelectric application. Due to the low thermal conductivity and high Seebeck coefficient feature of hybrid perovskites materials, they can be promising thermoelectric materials and hold the potential for the application of wearable energy generators and cooling devices. This mini-review will build a bridge between oxide perovskites and burgeoning hybrid halide perovskites in the research of thermoelectric properties with an aim to further enhance the relevant performance of perovskite-type materials.

## 1. Introduction

### 1.1. Thermoelectrics (TE)

**Figure 1:**
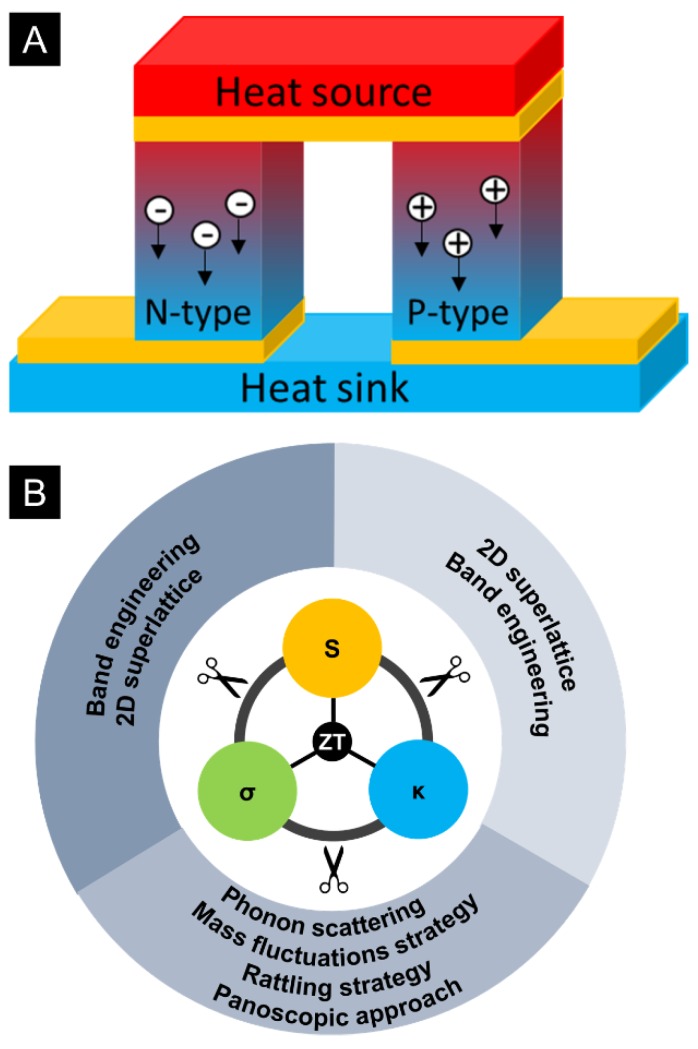
(**A**) Schematic of Seebeck effect; (**B**) schematic of interdependent relationship of Seebeck coefficient, electrical conductivity, and thermal conductivity, and the strategies to decouple their interdependency.

Thermoelectric effect includes Seebeck effect and Peltier effect. The Seebeck effect denotes the process that converts temperature gradient directly to electricity, which can be illustrated by the working principle of a thermoelectric generator (Figure 1A). In contrast, the Peltier effect can convert electrical energy to a temperature gradient, which leads to thermoelectric cooling device. Thermoelectric performance of a material is evaluated by the dimensionless thermoelectric figure of merit (ZT),

(1) $$\mathrm{ZT}=\frac{S^{2}\sigma }{\kappa }T$$

where S (V·K^−1^) is Seebeck coefficient, σ (S·m^−1^) is electrical conductivity, κ (W·m^−1^·K^−1^) is thermal conductivity, and T (K) is absolute temperature. S^2^σ is defined as the thermoelectric power factor. The thermoelectric energy conversion efficiency and the maximum cooling temperature for Peltier devices are dependent on the value of ZT [1].

The maximum efficiency (η) of a thermoelectric device is defined as the quotient of the energy provided to the load (W) and the heat energy consumed at the hot junction (Q). It is a function of ZT as well as the temperature of the hot and cold side (T_H_, T_C_), as shown in the equation

(2) $$\eta =\frac{W}{Q}=\frac{T_{H}-T_{C}}{T_{H}}\frac{\sqrt{1+\mathrm{ZT}}-1}{\sqrt{1+\mathrm{ZT}}+\frac{T_{H}}{T_{C}}}$$

Therefore, to enhance the thermoelectric properties of materials, namely increase the ZT value, the materials should have high Seebeck coefficient, high electrical conductivity, and low thermal conductivity [1,2,3,4].

However, simply enhancing the ZT value of TE materials is non-trivial, since the parameters are interrelated. One direct way to enhance the electrical conductivity of semiconductors is to increase the carrier concentration, as shown in the equation:

(3) $$\sigma =e\left(n_{e}\mu_{e}+n_{h}\mu_{h}\right)$$

where e is the elementary charge; n_e_ and n_h_ are the carrier concentrations of electrons and holes, respectively; μ_e_ and μ_h_ are the carrier mobility of electrons and holes, respectively.

However, the Seebeck coefficient for metal or degenerate semiconductor is defined by the equation:

(4) $$S=\frac{8\pi^{2}k_{B}^{2}}{3{\mathrm{eh}}^{2}}m^{*}T{\left(\frac{\pi }{3n}\right)}^{\frac{2}{3}}$$

where k_B_ is Boltzmann constant, h is Planck’s constant, m* is the effective mass of the charge carrier, T is absolute temperature, and n is the carrier concentration. According to Equation (4), S would decrease when increasing the carrier concentration.

The thermal conductivity is linked to electron and phonon contributions. The electronic thermal conductivity (electron contributions, κ_e_) can be expressed according to Wiedemann–Franz law:

(5) $$\kappa_{e}=L\cdot \sigma \cdot T$$

where L is the Lorentz number, and T is absolute temperature. Thus, it is directly related to electrical conductivity. As a consequence, the TE materials have an optimum carrier concentration to improve ZT value. The interdependent relationship was of σ, S, and κ were shown in Figure 1B, the ZT value was determined by σ, S, and κ, which was interconnected shown as the inner circle. To further enhance the ZT value, it is inevitable to decouple the interrelationship between the parameters. In inorganic TE materials, multiple ways have been applied to enhance the ZT value. For example, a phonon scattering mechanism was introduced by processing of superlattices and by accessing thermodynamically stable phase separation to suppress the lattice thermal conductivity without the expense of electrical conductivity [5,6,7]. Additionally, mass fluctuation strategy, rattling strategy, and the panoscopic approach were also used to decouple the relationship between σ and κ [7,8,9,10,11,12]. Energy filtering effect is also normally used to decouple the electrical conductivity and Seebeck coefficient, in which an energy barrier was introduced in the TE materials by nanocomposites or grain boundaries. This energy barrier would filter out low energy carriers and let high energy carriers pass. As a result, the Seebeck coefficient can be improved without suppressing the electrical conductivity [13,14,15,16]. Additionally, the band engineering, which is used to decouple the Seebeck coefficient and electrical conductivity, also includes degeneration of multiple valleys, electronic resonance states, synergistic nanostructuring, and highly mismatched isoelectronic doping. Furthermore, 2D superlattice can also be used to decouple S and σ [8,9,10,17,18]. These strategies were shown in the outer circle of Figure 1B.

TE devices can serve as generator and cooler and be used to recycle waste heat and manage temperature, respectively. Moreover, TE devices have advantages of no vibration, no noise, and highly reliable because they are solid-state devices without moving parts. In our previous studies, thick thermoelectric tellurium (Te) [19] and lead telluride (PbTe) [20] films were synthesized by electrodeposition with high film growth rate. The combination of electrochemical deposition of compound semiconductors with standard integrated circuit technique enables the fabrication of thermoelectric microdevices, which has a more compact size and a capability to handle a wider range of thermal and power management [21,22,23].

**Figure 2:**
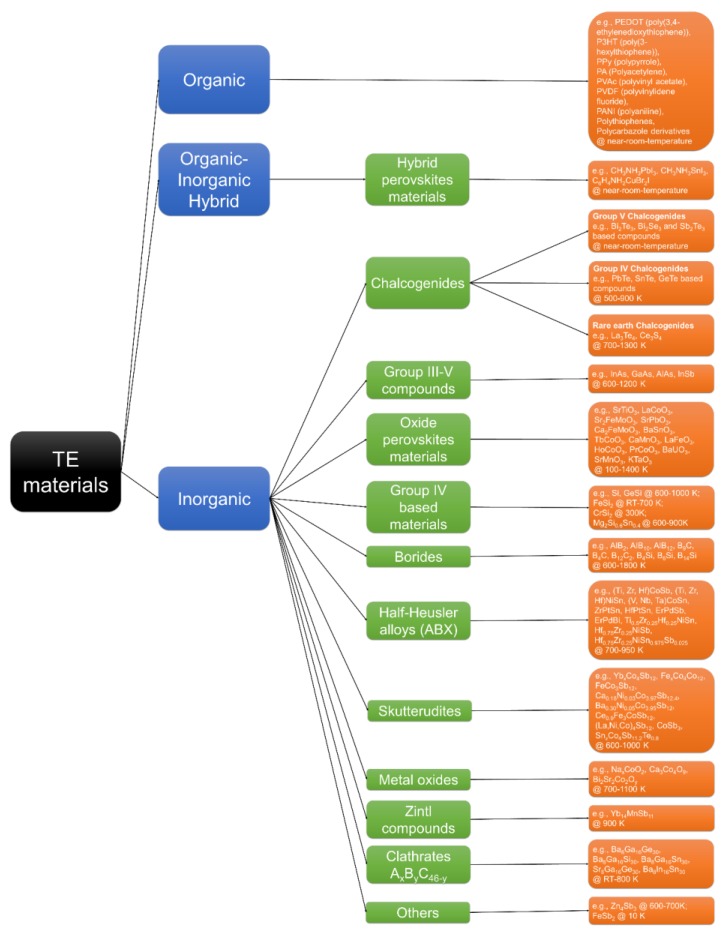
Thermoelectric materials studied through experimentation or calculation.

Materials, which are candidates for fabricating TE devices, can be classified as shown in Figure 2. Materials such as organic [5,6,24,25,26,27,28,29,30,31,32], hybrid perovskites [33,34,35] and the group V chalcogenides [3,7,10,11,12,36] are suitable for TE application at the near-room-temperature range. In addition, TE materials—such as the group IV chalcogenides [10,11,20,36], group III-V compounds [10,17,36], group IV-based materials [7,10,12,36], half-Heusler alloys [3,7,9,11], skutterudites [3,11,37], Zintl compounds [3,9,12,37], and clathrates [9,11]—are applicable at the middle temperature (about 400–900 K) range. Furthermore, materials—like rare earth chalcogenides [36], oxide perovskites [38,39,40], borides [36], and metal oxides [36]—can be used at a high temperature (>1000 K) range. However, normally there is no clear operation temperature boundary for each type of TE materials, for instance, rare earth chalcogenides, group III–V compounds, oxide perovskites materials, borides and metal oxides can be applied in a vast range of temperature depending on specific compounds [10,17,36,38,39,40]. The inorganic materials are the most well-studied TE materials [3,7,10,11,12,36,41]. We have studied the thermoelectric properties of PbTe films [20] and silver telluride nanofibers [41]. Additionally, Te nanostructures including nanowire [42], nanotree [42], and nanorice [43] were synthesized, which can be used to be embedded into other TE materials as nanocomposite [44,45,46] or converted to metal tellurides through cation exchange reaction [47,48]. Metal oxide materials, other than oxide perovskite materials, were investigated for TE application. For example, NaCo_2_O_4_ was reported to have an in-plane S of 100 µV·K^−1^ and σ of 5000 S·cm^−1^ at 300 K [49], Bi_2.2_Sr_1.8_Co_2_O_9_ achieved a ZT value of 0.19 at 973 K with S of ~150 µV·K^−1^, σ of ~82 S·cm^−1^ and κ of ~0.9 W·m^−1^·K^−1^ [50], Ca_3_Co_4_O_9_ showed a S of ~125 µV·K^−1^ and σ of ~28 S·cm^−1^ at 300 K [51], Zn_0.96_Al_0.02_Ga_0.02_O reached a ZT value 0.65 at 1247 K with S of 230 µV·K^−1^, σ of ~400 S·cm^−1^ and κ of 5 W·m^−1^·K^−1^ because of a bulk nanocomposite structure [52]. In the past decade, many efforts have been put into the investigation of organic thermoelectric materials [25,26,27,28,29]. Moreover, the hybrid perovskites started to draw attention for TE application [35]. In this review, we are going to focus on oxide and hybrid perovskite materials.

### 1.2. Perovskite Materials

Perovskite materials are any materials have the same type of crystal structure as calcium titanium oxide (CaTiO_3_). The general chemical formula for perovskites compounds is ABX_3_. A and B are two cations of very different sizes, and X is an anion that bonds to both [53]. Perovskite materials have a variety of applications, such as photovoltaics [53,54], light emitting diodes, thin film transistors, and so on [55]. Oxide perovskite materials have a long history of being applied for TE materials, while hybrid perovskite materials have just begun to be studied for TE applications in recent years.

**Figure 3:**
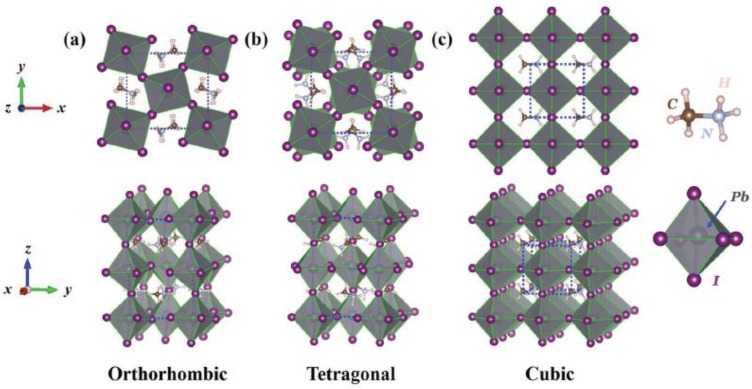
Atomistic configurations of (**a**) orthorhombic; (**b**) tetragonal; and (**c**) cubic phases of perovskite materials. Reproduced with permission [56]. Copyright 2016, Advanced Functional Materials.

The most common stable crystal phases of perovskite materials are orthorhombic, tetragonal, and cubic phases as shown in Figure 3 [56]. The stable phase of perovskites materials is determined by both ion size and temperature. For example, the orthorhombic phase of MAPbI_3_ is the low-temperature state and can keep its structure stable up to 165 K. With increased temperature, MAPbI_3_ undergoes a phase-transition to the tetragonal phase owing to the disordering of MA^+^. When temperature further reaches 327 K, highly disordered MA^+^ cations give rise to the high-symmetry state of MAPbI_3_ which has the cubic phase. The packing densities of the three phases follow the trend: c-MAPbI_3_ > o-MAPbI_3_ > t-MAPbI_3_, where the packing density of MAPbI_3_ increases as the crystal symmetry and temperature increases [56].

**Figure 4:**
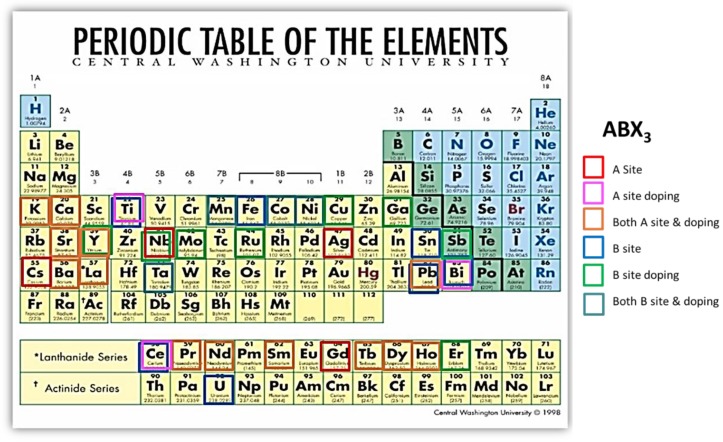
Elements that were used for perovskites in the A and B sites, which have been applied for thermoelectrics.

In this review, we will focus on the application of perovskite materials in thermoelectrics, which has not been systematically reviewed. The perovskite materials used for TE application can be classified into oxide and hybrid perovskites materials. The elements used in perovskite materials are summarized in Figure 4. The elements in the red box were used for A-site, the ones in the pink box were used for A-site doping, and the ones in the brown box were used for both A-site and A-site doping materials. The elements in the blue box were used for B-site, the ones in green box were used for B-site doping, and the ones in the cyan box were used for both B-site and B-site doping. Compared to the traditional materials used for TE application, such as metal chalcogenide materials like Bi_2_Te_3_ and PbTe, perovskite materials are less capitally expensive and can be processed by low energy cost methods, especially for hybrid perovskite materials, and can be used for flexible TE devices. The reported TE data about perovskite materials were shown in Table 1.

## 2. Oxide Perovskites

Oxide perovskite materials have been used for the application of thermoelectrics. Usually, oxide perovskite materials have relatively high Seebeck coefficient, low electrical conductivity, and high thermal conductivity at a specific temperature. For example, polycrystalline SrTiO_3_ has an S of −410 µV·K^−1^, a σ of 0.18 S·cm^−1^ and a κ of 7.5 W·m^−1^·K^−1^ at 375 K [59]. In general, two different approaches have been utilized to enhance ZT of thermoelectric materials. One approach is tuning the carrier concentration (n) to a suitable range since three interdependent thermoelectric parameters (i.e., S, σ, and κ) are functional to the carrier concentration [10]. The other approach is engineering of the material properties and structure of thermoelectric materials to decouple the parameters [16,163,164,165]. A variety of elements have been used for oxide perovskite materials, shown in Figure 4. In this review, the oxide perovskite materials were classified based on B-site elements, and their TE properties were discussed.

### 2.1. Titanium (Ti) as B-Site (ATiO_3_)

**Figure 5:**
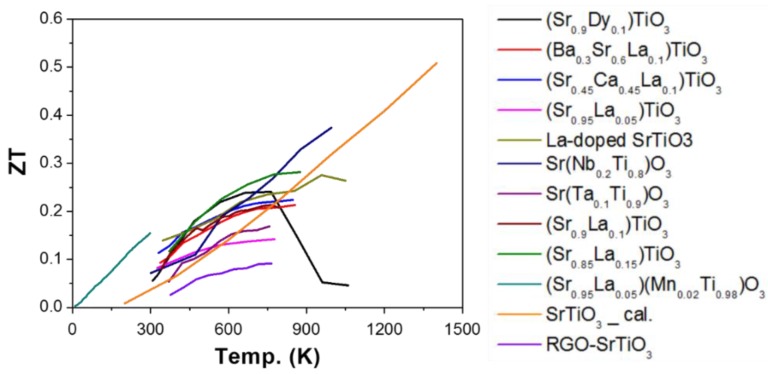
ZT value of ATiO_3_ based oxide perovskite materials, all the materials are n-type semiconductors. The symbol “cal.” means the results come from the calculation.

The reported ZT as a function of temperature, when Ti was used as B-site, was presented in Figure 5. All the materials are n-type thermoelectric materials.

#### 2.1.1. Enhancing Electrical Conductivity

To enhance the electrical conductivity of ATiO_3_, chemical doping is applied. Although the electrical conductivity, Seebeck coefficient, and thermal conductivity are interdependent, it is necessary to tune the carrier concentration to its optimum range. The electrical conductivity was modified by oxygen vacancy [38,57,58,59], and chemical substitution in A-site [40,60,61,62,63,64,65,66,67] and B-site [65,68].

**Figure 6:**
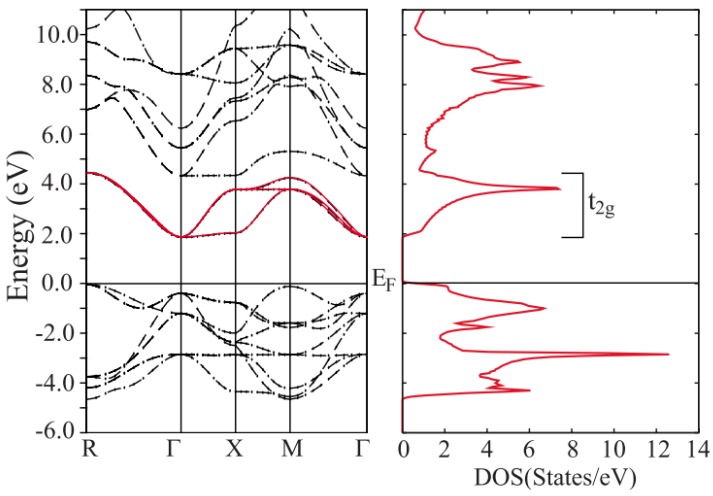
The band structure and the density of states of SrTiO_3_ [80]. Copyright 2010, Physical Review B.

Oxygen vacancy has been induced to enhance the electrical conductivity. The perovskite-type ATiO_3_ is a wide gap band-insulator, where the valence band comes from the oxygen 2p state and the conduction band from the Ti 3d-t2g state with threefold orbital degeneracy [58], Figure 6 shows the band structure and the density of states of SrTiO_3_ (STO) [80]. The oxygen vacancy, which can be introduced into perovskite materials by controlling oxygen partial pressure, hydrogen reduction, and reduced graphene oxide, can generally add two electrons in the Ti site, providing charge carriers, therefore enhancing the electrical conductivity [38,57,58,59]. For example, Onoda et al. investigated the perovskite-type oxygen deficient system SrTiO_3−δ/2_ with 0.0046 ≤ δ < 0.06, the carrier concentration was enhanced from 2.5 × 10^18^ to 1.0 × 10^21^ cm^−3^, when δ increased from 0.0046 to 0.06, resulting in the enhancement of electrical conductivity from about 5 to 1000 S·cm^−1^. The power factor 2000 µW·m^−1^·K^−2^ was obtained with the carrier concentration of 2 × 10^20^ cm^−3^ [58]. Choi et al. investigated La-doped SrTiO_3_ thin films with controlled elemental vacancies by varying the oxygen partial pressure P(O_2_) during the film growth [38]. Decreasing P(O_2_) would increase the cation (La and Sr) and oxygen vacancies [38]. The cation vacancies are known to compensate the generation of carriers by the oxygen vacancies [38]. Also, it also expands the lattice, thereby disturbing the carrier transport. The carrier concentration was tuned from ~0.43 × 10^21^ to 1.15 × 10^21^ cm^−3^ when oxygen partial pressure decreased from 10^−2^ to 10^−6^ Torr [38]. Seebeck coefficient of the La-doped SrTiO_3_ thin films can be modulated from −120 to −260 µV·K^−1^ [38]. Ravichandran et al. reported that the oxygen vacancy can tune the carrier concentration from 1 × 10^21^ to 3 × 10^21^ cm^−3^ at La doping level of 15% in La-doped SrTiO_3_, and a ZT value of 0.28 was achieved at 873 K at a carrier concentration of 2.5 × 10^21^ cm^−3^ [57]. Feng et al. induced the oxygen vacancy into undoped SrTiO_3_ (STO) by hydrogen reduction (STO-H) and reduced graphene oxide (RGO). Their results showed that only 0.64 vol % of graphene content would enhance electrical properties of STO significantly, where the carrier concentration was 3.64 × 10^18^, 4.54 × 10^19^, and 1.59 × 10^20^ cm^−3^ for pure STO, STO-H, and RGO/STO, respectively. Additionally, the electrical conductivity increased from 2 to 14 to 30 S·cm^−1^ at 760 K, when the sample changed from pure STO to STO-H to RGO/STO. Electron energy loss spectrum confirmed high oxygen vacancy concentration in the RGO rich area in RGO/STO composite. The ZT value was increased to 0.09 at 760 K for RGO/STO composite, compared to 0.008 for pure STO [59]. Additionally, Lee et al. reported that electrical conductivity of BaTiO_3_ can reach 11 S·cm^−1^ at 300 K with a carrier concentration of ~3 × 10^20^ by inducing oxygen vacancy [79], while the single crystal BaTiO_3_ shows electrical conductivity of 0.05 S·cm^−1^ at 300 K [70]. However, the Seebeck coefficient decreased to −280 µV·K^−1^ [79], compared to −550 µV·K^−1^ at 300 K for single crystal BaTiO_3_ [70]. In short, the oxygen vacancy can increase the carrier concentration of the ATiO_3_, thus improving the electrical conductivity. Additionally, oxygen vacancy can also reduce the lattice thermal conductivity because of phonon scattering [79]. However, the strategy of introducing oxygen vacancy needs to cooperate with other types of doping or strategies to achieve relatively high ZT value.

Additionally, chemical substitution (such as La [60,61,62,63,64,65,66,67,74], Ce [66], Ba [39,72], Pr [40], and Y [71]) in A-site is a widely-used method to enhance the electrical conductivity of ATiO_3_. La is the most widely studied A-site doping element in SrTiO_3_. The carrier concentration of Sr_1−x_La_x_TiO_3_ increases from 2.0 × 10^18^ to 9.0 × 10^21^, when x increased from 0 to 0.5. As a result, the electrical conductivity is enhanced from 0.3 to 2000 S·cm^−1^ [60,61,62,63,64,65,66,74]. Since the electrical conductivity is interrelated with Seebeck coefficient, heavily La-doped STO also has a relatively low Seebeck coefficient. Additionally, La doping does not necessarily increase the thermal conductivity, which is probably attributed to the decrease of the mean free path of the phonons caused by lattice defects [63]. As a result, the ZT value achieved for Sr_0.95_La_0.05_TiO_3_ (La-doped STO) was 0.14 at 773 K with n of about 8 × 10^20^ cm^−3^ and σ of 150 S·cm^−1^ [63]. Theoretical calculation by Boltzmann transport theory predicted that a ZT value of 0.7 at 1400 K could be achieved with an n of 4 × 10^20^ cm^−3^ for La-doped STO [85]. However, such a high ZT value has not been obtained experimentally. Additionally, La was also used to dope STO together with Ba (i.e., Sr_0.9−x_Ba_x_La_0.1_TiO_3_) [72] and oxygen vacancy [57]. The electrical conductivity of Sr_0.9−x_Ba_x_La_0.1_TiO_3_ increased from 200 to 2500 S·cm^−1^, when x increased from 0 to 0.9 at 420 K [72]. This is caused by the decrease of the Ti–Ti distance, which increases the overlap of Ti 3d orbital in the conduction band [72]. The highest ZT value achieved in the study is 0.13 at 420 K, with a formula of (Ba_0.3_Sr_0.6_La_0.1_)TiO_3_ [72]. Moreover, when the La content increased from 0 to 0.15, the carrier concentration of oxygen vacancy doped STO increased from ~3 × 10^20^ to 3 × 10^21^ cm^−3^, and electrical conductivity from ~70 to 700 S·cm^−1^ [57]. The highest ZT value of 0.28 was achieved at 873 K at a carrier concentration of 2.5 × 10^21^ cm^−3^ [57]. Furthermore, Pr was also used as A-site dopant in Sr_1−x_Pr_x_TiO_3_ (0 < x < 0.125), in which, a linear increase in carrier concentration is observed with increasing Pr concentration [40]. The carrier concentration increased from 0.3 × 10^21^ cm^−3^ to 2.1 × 10^21^, when x changed from 0 to 0.125 [40], and the electrical conductivity was enhanced from 250 to 700 S·cm^−1^, when x changed from 0.05 to 0.125 [40]. The mobility of STO was enhanced to about 12 cm^2^·V^−1^· s^−1^ [40], compared to 4–8 cm^2^·V^−1^·s^−1^ [166,167,168,169], in which the dramatic enhancement in carrier mobility was attributed to the formation of Pr-rich grain boundaries [40]. A ZT value of 0.4 at 773 K was achieved attributed to the high mobility [40]. In short, the combination of chemical doping and oxygen vacancy can lead to relatively high ZT value [57]. Furthermore, Pr doping with Pr-rich boundary can achieve high carrier concentration and mobility, which resulted in the highest ZT value obtained experimentally so far for ATiO_3_ [40].

Furthermore, chemical doping in B-site was applied to enhance the electrical conductivity. Nb [65,68,69,76], Ta [78], Mn [83] and Co [89] was used as B-site dopant in SrTiO_3_. Nb is the most widely used B-site dopant (Sr(Nb_x_Ti_1−x_)O_3_, 0.01 < x < 0.4), in which substituted Nb^5+^ at Ti^4+^ site generates carrier electrons [68]. In the doping range, the carrier concentration was tuned from ~0.1 × 10^21^ to 7 × 10^21^ cm^−3^, leading to electrical conductivity varying from ~95 to 353 S·cm^−1^ [65,68,69,76]. Also, the Nb-doped SrTiO_3_ film has a large Seebeck coefficient. For example, a rather large |S| (i.e., 160 μV·K^−1^) is observed for the 40% Nb-doped SrTiO_3_ film in spite of an extremely high carrier concentration (i.e., 7 × 10^21^ cm^−3^) [68]. It is worth mention that |S| of n-type lead telluride (PbTe) is less than 50 μV·K^−1^ in the carrier concentration range of 10^20^ cm^−3^ [16,170]. The large |S| values in high carrier concentration films result from the gradual increase in effective mass (m*/m^0^, from 7 to 11) [65,68]. The enhancement of m*/m^0^ is owing to an increase in the lattice parameter or the distance between two neighboring Ti ions, leading to the decrease in overlapping between Ti 3d-t2g orbitals [65,68]. Moreover, Nb substitute can also suppress the thermal conductivity of STO [77]. A ZT value of ~0.35–0.37 at 1000 K was achieved for 20% Nb-doped STO [68,76]. Additionally, the theoretical analysis by advanced ab initio calculations showed that superlattice in Nb-doped STO could remarkably improve the Seebeck coefficient compared to the bulk at equivalent doping concentration, and the Seebeck coefficient in similar heterostructures would be improved more by weak, rather than tight, spatial confinement [82]. Moreover, the slight Mn substitution in B-site of Sr_1−x_LaxTiO_3_ can also enhance the effective mass of electrons, giving rise to an increase in |S| [83]. For example, the S was enhanced from −120 to −180 μV·K^−1^, and the ZT value was changed from 0.07 to 0.15 at 300 K when the composition is tuned from Sr_0.95_La_0.05_TiO_3_ to Sr_0.95_La_0.05_Ti_0.96_Mn_0.04_O_3_ [83]. This phenomenon can be attributed to the coupling between itinerant electrons and localized spins or coupling between itinerant electrons and local lattice distortion around Mn^3+^ ions [83]. Additionally, as Ta and Co-doped STO, the ZT value was 0.17 at 752 K and 0.135 at 300 K for SrTi_0.9_Ta_0.1_O_3_ [78] and SrTi_0.875_Co_0.125_O_3_ [89], respectively.

In short, the B-site doping cannot tune the carrier concentration as efficiently as A-site doping, but it can increase the effective mass of the materials, therefore enhancing the Seebeck coefficient at high carrier concentrations. In perspective, it can be promising to introduce both A-site and B-site doping into the materials, where A-site improves the electrical properties and B-site enhances the Seebeck coefficient.

#### 2.1.2. Decoupling Electrical Conductivity and Seebeck Coefficient

The Seebeck coefficient is negatively interrelated with electrical conductivity (Equations (3) and (4)), where Seebeck coefficient decreases with increasing carrier concentration. However, there are some strategies that can enhance the Seebeck coefficient without reducing electrical conductivity. For example, the existence of oxygen vacancy can change the density of electronic states around the Fermi energy, which results in larger Seebeck coefficient [62,63,64]. The Seebeck coefficient of oxygen-deficient samples (e.g., S of −300 μV·K^−1^ at 750 K for Sr_0.9_La_0.1_TiO_3−δ_) are larger than those with near-stoichiometric ones with the same La doping level (e.g., S of −255 μV·K^−1^ at 750 K for Sr_0.9_La_0.1_TiO_3_) [62,63]. This superiority leads to higher ZT value of 0.21 at 750 K [64]. Moreover, B-site substitution of Ti by Nb [65,68,76] and Mn [83] can also enhance the Seebeck coefficient by increasing the effective mass of electrons. A |S| of 160 μV·K^−1^ is obtained from the 40% Nb-doped SrTiO_3_ film despite a high carrier concentration of 7 × 10^21^ cm^−3^ [65,68,76]. A-site substitution of Sr by La in SrTiO_3_ (i.e., Sr_1−x_La_x_TiO_3_, 0.02 ≤ x ≤ 0.1) was also reported to have relatively large Seebeck coefficient compared to the materials in the same carrier concentration range of 10^21^ cm^−3^, which is attributed to the orbital degeneracy of the Ti 3d-t2g conduction band, the relatively large electron effective mass, and large energy-dependent scattering rate [61,74]. Additionally, Sun et al. investigated the TE properties of La-doped SrTiO_3_ and Ba doped KTaO_3_ through the first-principles band calculation. Their results showed that the Seebeck coefficients are higher in multiple band systems than those with single-band [80], since the Fermi level tends to stay lower for systems with multiple bands given a fixed number of doped electrons [80]. Furthermore, quantum confinement effect can effectively enhance the Seebeck coefficient of SrTiO_3_. Ohta et al. reported a high-density two-dimensional electron gas (2DEG) confined within 0.5 to 16 unit cell layer thickness in SrTiO_3_ (i.e., SrTiO_3_/SrTi_0.8_Nb_0.2_O_3_/SrTiO_3_ quantum-well, where n_e_ = 10^15^ cm^−3^ for SrTiO_3_ and n_e_ = 2.4 × 10^21^ cm^−3^ for SrTi_0.8_Nb_0.2_O_3_) [75]. A dramatic increase in |S| at room temperature is observed when the thickness of the SrTi_0.8_Nb_0.2_O_3_ layer becomes less than 1.56 nm (i.e., a four-unit cell layer thickness) [75]. For example, the one unit cell layer thickness of the SrTi_0.8_Nb_0.2_O_3_ reached a large |S| of 480 µV·K^−1^ at 300 K, approximately five times larger than that of SrTi_0.8_Nb_0.2_O_3_ bulks (i.e., 108 µV·K^−1^ at 300 K with n_e_ of 2.4 × 10^21^ cm^−3^) [75]. This significant improvement of |S| suggests that strong electron confinement can be achieved by using strongly directive Ti 3d (t2g) orbitals; direct overlap between neighboring 3d orbitals is relatively small, and carrier electrons tend to localize in the Ti atoms [75]. They also claimed that with the measured |S| of 850 µV·K^−1^, estimated σ_2DEG_ of 1.4 × 10^3^ S·cm^−1^, and κ of 12 W·m^−1^·K^−1^ from bulk single-crystal SrTiO_3_, an estimated ZT of 2.4 was observed, corresponding to ZT~0.24 for a complete device having the 2DEG as the active region [75].

#### 2.1.3. Reducing Thermal Conductivity

The thermal conductivity is correlated with electrical conductivity because of electron contributions (Equation (5)). To decouple to κ and σ, suppressing the phonon contribution (lattice thermal conductivity, κ_l_) is usually applied, which was realized in ATiO_3_ by grain boundary phonon scattering [59], point defect phonon scattering [73], distortion of crystal lattice [62,77,83], and so on.

For instance, the lattice thermal conductivity of RGO/STO is 3.7 W·m^−1^·K^−1^ at 760 K, which is 37% lower than that of pure STO (i.e., 5.9 W·m^−1^·K^−1^ at 760 K) [59]. This can be ascribed to the highly restrained grain size in RGO/STO, which is 20 times smaller than that of pure STO [59].

Additionally, Ca and rare earth elements substitution in A-site can suppress the κ_l_. Ca was used to substituted Sr in Sr_0.9−x_Ca_x_La_0.1_TiO_3_ (0 < x < 0.6), and the resulted showed that the thermal conductivity decreased from 4.7 to 3.3 W·m^−1^·K^−1^ when x increased from 0 to 0.6, which is caused by the point defect scattering and the lowered symmetry [73]. As a result, Sr_0.45_Ca_0.45_La_0.1_TiO_3_ has a ZT value of 0.22 at 873 K, compared to 0.2 for Sr_0.9_La_0.1_TiO_3_ at 873 K [73]. Muta et al. studied the rare earth (e.g., Y, La, Sm, Gd, and Dy) doped SrTiO_3_ [62]. The thermal conductivity of SrTiO_3_ is dependent on the doping element, namely: the thermal conductivity decrease monotonically with the ionic rare earth radius, in which it decreased from 4.3 to 2.6 W·m^−1^·K^−1^ when the dopant changed from La to Dy [62]. This phenomenon is caused by lattice distortion with difference ionic radii, leading to phonon scattering [62]. The enhancement of ZT value in this work is owing to reducing the thermal conductivity without deteriorating the electrical conductivity of SrTiO_3_, and (Sr_0.9_Dy_0.1_)TiO_3_ showed a ZT value of 0.22 at 573 K [62]. Moreover, Eu^2+^ substitution of SrTi_0.8_Nb_0.2_O_3_ can reduce the thermal conductivity without reducing the thermoelectric power factor [77]. The mean free path of the phonon was reduced about 12% at room temperature when half of the Sr^2+^ was substituted by Eu^2+^ [77]. As a result, the κ of the SrTi_0.8_Nb_0.2_O_3_ ceramic at room temperature is 8.8 W·m^−1^·K^−1^, while it decreased to 7.7 W·m^−1^·K^−1^ with 50% of the Sr was substituted by Eu [77].

Also, the slight Mn substitution with some defects in B-site of Sr_1−x_LaxTiO_3_ can enhance anharmonic lattice vibrations resulting in inelastic phonon-phonon scattering, which effectively suppresses thermal conductivity at high electrical conductivity [83]. The thermal conductivity was reduced from 7.1 to 3.8 W·m^−1^·K^−1^, when the composition changed from Sr_0.95_La_0.05_TiO_3_ to Sr_0.95_La_0.05_Ti_0.98_Mn_0.02_O_3_ [83]. The ZT value of Sr_0.95_La_0.05_Ti_0.98_Mn_0.02_O_3_ reached 0.15 at 300 K compared to 0.07 for Sr_0.95_La_0.05_TiO_3_ [83].

In short, among all the strategies (i.e., inducing grain boundary and chemical substitution in A-site and B-site), the most effective strategy to suppress lattice thermal conductivity is by rare earth substitution of A-site [62] and Mn substitution of B-site [83].

### 2.2. Manganese (Mn) as B-Site (AMnO_3_)

**Figure 7:**
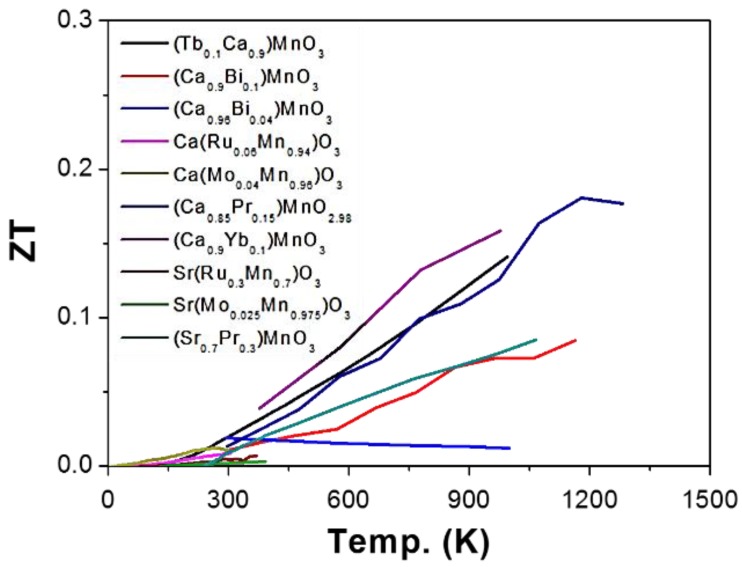
ZT value of AMnO_3_ based oxide perovskite materials, all the materials are n-type semiconductors.

The thermoelectric properties of AMnO_3_ were summarized, and the ZT values as a function of temperature were shown in Figure 7, where all the materials are n-type semiconductors.

#### 2.2.1. Enhancing Electrical Conductivity

Chemical doping in A-site was used as a strategy to enhance the electrical conductivity. Multiple elements have been used as A-site dopant in AMnO_3_, including In [91], Sn [91], Sb [91], Pb [91], Bi [91,103], and rare earth element (e.g., Yb, Y, La, Ce, Sm, Dy, Tb, Ho, and Pr) [90,91,92,93,94,95,96,97]. In general, the electrical conductivity increases as the ionic radius (r_A_) decreases [95]. The overlapping of Mn and O orbitals are substantially influenced by the r_A_, which determines the Mn–O–Mn bond angles [95]. The decrease of r_A_ enhances electrical conductivity owing to the strength of the bending of the Mn–O–Mn bond, which narrows the electron conduction bandwidth [95]. Additionally, a relatively high ZT value can be achieved not necessarily at the highest doping level, but sometimes at an optimum doping level. For example, the electrical conductivity of Ca_1−x_Pr_x_MnO_2.98_ (0 < x < 0.05, 0.1, 0.15) increased from 40 to 125 S·cm^−1^ at 1100 K, when x changed from 0 to 0.15, leading to a ZT value of 0.17 at 1100 K for Ca_0.85_Pr_0.15_MnO_2.98_ [94]. The electrical conductivity increased from ~67 to 333 S·cm^−1^ at 1073K for Sr_x_Pr_1−x_MnO_3_, when x changed from 0 to 0.7 [97]. The electrical conductivity of (Ca_1−x_R_x_)MnO_3_ (R: Tb, Ho, Y) increase with x value (e.g., from 100 to 182 S·cm^−1^ for (Ca_1−x_Tb_x_)MnO_3_, from 91 to 250 S·cm^−1^ for (Ca_1−x_Ho_x_)MnO_3_ when x increased from 0.1 to 0.3), and the highest ZT value was achieved at x = 0.1 for all three materials (e.g., 0.13, 0.08, 0.15, for (Tb_0.1_Ca_0.9_)MnO_3_, (Ho_0.1_Ca_0.9_)MnO_3_), and (Y_0.1_Ca_0.9_)MnO_3_, respectively) [90]. The electrical conductivity of Ca_1−x_Bi_x_MnO_3_ increased with Bi doping from 32 S·cm^−1^ at x = 0.02 to 222 S·cm^−1^ at x = 0.2, and a ZT value of 0.086 was achieved when X = 0.04 (i.e., Ca_0.96_Bi_0.04_MnO_3_) at 1000 K [103]. A ZT value of 0.16 was obtained for Ca_0.9_Yb_0.1_MnO_3_ with σ of 167 S·cm^−1^ at 1000 K [95].

On the other hand, chemical doping in the B-site was studied to enhance the electrical conductivity. Multiple elements have been used as a B-site dopant in AMnO_3_, including Mo [104,110], Ru [104,108], Nb [105,107], Ta [105], and Ni [100]. The electrical conductivity of CaMn_0.96_Mo_0.04_O_3_ was measured to be about 154 S·cm^−1,^ and the ZT value is 0.012 at 270 K [104]. The electrical conductivity was 67 S·cm^−1^ for SrMn_0.7_Ru_0.3_O_3_, and the ZT value reached 0.01 at 370 K [108]. CaMn_0.96_Ta_0.04_O_3_ reached an electrical conductivity of 29 S·cm^−1^ and a ZT value of 0.05 at 1000 K [105]. CaMn_0.98_Nb_0.02_O_3_ have a S of −240 µV· K^−1^, σ of 31 S·cm^−1^ and κ of 0.8 W·m^−1^·K^−1^ at 1070 K, leading a ZT values of 0.32 at 1070 K [107]. However, the significant enhancement of ZT value to 0.32 was mainly attributed to low thermal conductivity, caused by phonon scattering from the nanosized twinned domains or the porous morphology [107]. In short, the B-site doping can also serve the purpose of improving the electrical conductivity, but it is not as effective as A-site doping.

#### 2.2.2. Reducing Thermal Conductivity

The thermal conductivity of AMnO_3_ can be decoupled from electrical conductivity by introducing phonon scattering, which was realized by grain boundary [107], lattice distortion [94,95,102], and orbital fluctuation in the paramagnetic phase [110].

For example, CaMn_0.98_Nb_0.02_O_3_ has a κ of 0.8 W·m^−1^·K^−1^ at 1070 K, caused by higher phonon scattering from the nanosized twinned domains or the porous morphology, in which a ZT value of 0.32 at 1070 K was obtained [107]. Additionally, chemical substitution can result in local lattice distortions linked with quasi-static Mn^3+^ ions, therefore suppressing the lattice thermal conductivity [94,95,102]. For instance, κ of Ca_1−x_Pr_x_MnO_2.98_ decreased from 2.1 to 1.5 W·m^−1^·K^−1^ at ~1100 K, when x varied from 0 to 0.15 [94]; κ of CaMn_1−x_Mo_x_O_3_ decreased from 2.7 to 1.3 W·m^−1^·K^−1^ at 300 K, when x increased from 0.07 to 0.15 [102]; κ of CaMnO_3_ decreased from 3.6 to ~1.7 W·m^−1^·K^−1^ at 300 K, when Ca was substituted with Yb [95] and Ho [95]. Moreover, Mo in Sr(Mn_1−x_Mo_x_)O_3_ introduces orbital fluctuation in the paramagnetic phase, leading to scattering the acoustic phonons, which results in a reduction of the κ from 6.1 to 5 W·m^−1^·K^−1^ at 390 K, when x increased from 0 to 0.025 [110].

In short, thermal conductivity is dominated by lattice thermal conductivity for AMnO_3_ [107,110], and the results showed that phonon scattering by nano-engineering is relatively more effective at suppressing the lattice thermal conductivity [107].

### 2.3. Cobalt (Co) as B-Site (ACoO_3_)

**Figure 8:**
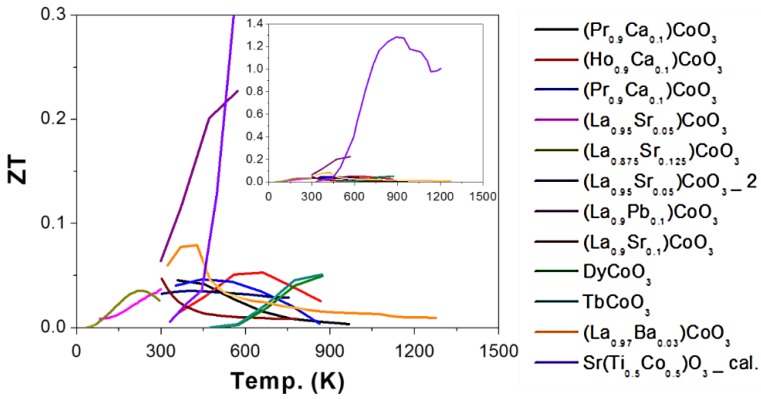
ZT value of ACoO_3_ based oxide perovskite materials, all the materials are p-type semiconductors. The symbol “cal.” means the results come from the calculation.

The reported ZT as a function of temperature when Co was used as B-site (ACoO_3_) was presented in Figure 8. Normally, the rare earth elements (i.e., Pr [113,114,115], Ho [114,121], Nd [113,114,115], Tb [115], Sm [113,114], Gd [113,114], Dy [114,115], and La [122,123,124,125,126,127,128,129]) were used as A-site elements. The electrical conductivity increased with increasing ionic radii of the rare earth cations. The degree of distortion of the structure determines the onset of the electronic localization-delocalization transition. With a given R^3+^ size, the CoO_6_ octahedron is tilted in order to optimize R-O bond distances, resulting in a deviation of the Co-O-Co angles from 180°, which determines the degree of overlapping of the cobalt 3d and oxygen 2p orbitals. Large rare-earth ions cause the Co-O-Co bonds to straighten out and become closer to 180°. The Co 3d and O 2p orbital overlap increases as the Co-O-Co angles become closer to 180°, and this structural change leads to the observed rare-earth element dependence of the electronic behavior of RCoO_3_ [113,114,115].

For example, Hashimoto et al. reported Pr, Nd, Tb, and Dy as A-site in cobalt oxide RCoO_3_ (R = Pr, Nd, Tb, Dy) ceramics [115]. The electrical conductivity increased with increasing ionic radii of the rare earth cations (ionic radii: Pr^3+^ > Nd^3+^ > Tb^3+^ > Dy^3+^), where the electrical conductivity increased from 180 to 400 S·cm^−1^ at 873 K, when A-site changed from Dy (DyCoO_3_) to Pr (PrCoO_3_) [115]. However, the highest ZT value of 0.05 was obtained for DyCoO_3_ at 873 K with S of about 80 µV·K^−1^ and κ of 1.6 W·m^−1^·K^−1^ [115]. Moon et al. presented the TE properties of (R_0.9_Ca_0.1_)CoO_3_ (R = Gd, Sm, Nd, Pr) [113]. The results showed that electrical conductivity of samples increased sharply with increasing the ionic radius of the rare-earth element (Pr > Nd > Sm > Gd). The σ changed from 23 to 210 S·cm^−1^, when the samples changed from (Gd_0.9_Ca_0.1_)CoO_3_ to (Pr_0.9_Ca_0.1_)CoO_3_ [113]. A ZT value of 0.047 was achieved at 358 K for (Pr_0.9_Ca_0.1_)CoO_3_ with σ of 210 S·cm^−1^ and S of about 108 µV·K^−1^ [113].

In addition, similar phenomenon were observed when the ionic radius of dopants (Ca^2+^ < Sr^2+^ < Ba^2+^) increased for (Pr_0.9_M_0.1_)CoO_3_ (M = Ca, Sr, Ba). The electrical conductivity increased from 350 to 420 S·cm^−1^, when varied from (Pr_0.9_Ca_0.1_)CoO_3_ to(Pr_0.9_Ba_0.1_)CoO_3_ [114].

Furthermore, the electrical conductivity of LaCoO_3_ normally increases with increasing temperature. For instance, σ increased from 14 to 1230 S·cm^−1^ for La_0.95_Sr_0.05_CoO_3_, when temperature increased from 300 to 750 K) [124]. This temperature dependent conduction behaviors of La_0.95_Sr_0.05_CoO_3_ were explained regarding small polaron hopping mechanism with positive polarons ${\left({\mathrm{Co}}^{4+}{}_{{\mathrm{Co}}^{3+}}\right)}^{\cdot }$ as transport carriers [124]. Increase of σ with increasing temperature was attributed to the increase of the ${\left({\mathrm{Co}}^{4+}{}_{{\mathrm{Co}}^{3+}}\right)}^{\cdot }$ concentration on the Co sites [124].

#### 2.3.1. Chemical Doping in A-Site

The strategy of A-site doping was applied to tune the electrical conductivity further. LaCoO_3_ was investigated with Sr [122,123,124,125], Pb [126], Na [126], and Ba [128] used as A-site dopant. Sr-doped LaCoO_3_ (i.e., La_1−x_Sr_x_CoO_3_, 0 ≤ x ≤ 0.2) was investigated_._ The electrical conductivity enhanced from ~2 to 296 S·cm^−1^, when x changed from 0 to 0.2 at 300 K [123,124,125]. A ZT value of La_0.9_Sr_0.1_CoO_3_ obtained was 0.046 at 300 K with σ of ~180 S·cm^−1^, S of ~120 µV·K^−1^, and κ of 1.45 W·m^−1^·K^−1^ [125]; and a ZT value of 0.18 was achieved for La_0.95_Sr_0.05_CoO_3_ with S of ~710 µV·K^−1^ and κ of 3.7 W·m^−1^·K^−1^ [123]. Pb and Na were used as A-site dopant for LaCoO_3_ with composition La_0.9_R_0.1_CoO_3_ (R = Pb, Na). The electrical conductivity for undoped polycrystalline LaCoO_3_ was 25 S·cm^−1^, and was enhanced to 250 and 33 S·cm^−1^ at 575 K, when doped with Pb (i.e., La_0.9_Pb_0.1_CoO_3_) and Na (i.e., La_0.9_Na_0.1_CoO_3_), respectively [126]. The thermal conductivity of LaCoO_3_, La_0.9_Pb_0.1_CoO_3_, and La_0.9_Na_0.1_CoO_3_ were 1.83, 1.55, and 0.9 W·m^−1^·K^−1^, respectively [126]. A ZT value of 0.23 was achieved at 575 K for La_0.9_Pb_0.1_CoO_3_ with S of ~110 µV·K^−1^, which was attributed to both increasing the electrical conductivity and suppressing the lattice thermal conductivity [126]. The electronic part of the thermal conductivity contribution was estimated using the Wiedemann–Franz law in La_1−x_R_x_CoO_3_ to be about 1% in LaCoO_3_, 3% in La_0.9_Na_0.1_CoO_3_, and 31% in La_0.9_Pb_0.1_CoO_3_ of the total thermal conductivity at 575 K, which shows that lattice thermal conductivity plays a major role in these samples [126]. The overall suppression of the total thermal conductivity upon Pb and Na substitution mainly comes from the reduction of the lattice thermal conductivity, possibly through the lattice strain induced by the atomic disorder at the A-site of the perovskite structure and the mixed valency at the Co-site [126].

Additionally, Moon et al. investigated TE properties of (Ho_1−x_Ca_x_)CoO_3_. The electrical conductivity was enhanced from 1 × 10^−4^ S·cm^−1^ to 2 S·cm^−1^ when x changed from 0 to 0.1 at 573 K. The highest ZT value obtained was 0.051 at 573 K for Ho_0.9_Ca_0.1_CoO_3_ with S of about 210 µV·K^−1^ and κ of 0.75 W·m^−1^·K^−1^ [121].

#### 2.3.2. Chemical Doping in B-Site

Mn [127] and Ni [129] were used as a B-site dopant in LaCoO_3_. For example, the electrical conductivity reached 5 S·cm^−1^ at 400 K for LaCo_0.99_Mn_0.01_O_3_ [127]. The carrier concentration increases from 6.1 × 10^14^ to 2.6 × 10^17^ cm^−3^ for YCo_1−x_Ni_x_O_3_, leading to increasing in electrical conductivity from 0.0015 to 0.011 S·cm^−1^ at room temperature, when x changes from 0 to 0.07 [129].

#### 2.3.3. Reducing Thermal Conductivity

Suppressing the lattice thermal conductivity was applied to decouple σ and κ, which was realized by chemical substitution. The chemical substitution can induce lattice disorder, lattice strain, or mass disorder, leading to phonon scattering [122,126,128]. In the reported studies, the effect of A-site doping (e.g., Sr [122], Pb [126], Na [126], Ba [128]) on lattice thermal conductivity of ACoO_3_ was investigated, while the B-site doping was barely discussed.

For instance, single crystal La_0.82_Sr_0.18_CoO_3_ has a lower κ of 1 W·m^−1^·K^−1^ compared to ~5 W·m^−1^·K^−1^ for LaCoO_3_ at 60 K, due to lattice disorder caused by temperature and/or doping-induced spin-state transitions of the Co ions [122]. Moreover, the thermal conductivity of LaCoO_3_ can be tuned by Na and Pb substitution of La. The thermal conductivity of LaCoO_3_, La_0.9_Pb_0.1_CoO_3,_ and La_0.9_Na_0.1_CoO_3_ were 1.83, 1.55, and 0.9 W·m^−1^·K^−1^, respectively, which may be attributed to the lattice strain induced by the atomic disorder at the A-site of the perovskite structure and the mixed valency at the Co-site [126]. The thermal conductivity of LaCoO_3_ can also be suppressed by Ba substitution. The thermal conductivity of La_1−x_Ba_x_CoO_3_ (x = 0.01, 0.03, 0.05) was about 0.5–0.6 W·m^−1^·K^−1^ at 320 K [128], while the thermal conductivity of LaCoO_3_ is about 2.5 W·m^−1^·K^−1^ in the same temperature range [126]. This phenomenon can be explained by mass disorder scattering of phonons, reducing the lattice part of the thermal conductivity, resulting in a ZT value of 0.08 in the 400 K range for La_0.97_Ba_0.03_CoO_3_ with σ of 350 S·cm^−1^ and S of ~220 µV·K^−1^ [128].

In short, the A-site dopant is more effective than B-site dopant in improving the carrier concentration and electrical conductivity. However, high ZT of 0.23 was achieved with the combination of high electrical conductivity and low thermal conductivity [126].

### 2.4. Other Elements, Including Iron (Fe), Nickel (Ni), Tin (Sn), Lead (Pb), Bismuth (Bi), Molybdenum (Mo), Ruthenium (Ru), and Uranium (U) as B-Site (ABO_3_)

#### 2.4.1. Fe as B-Site (AFeO_3_)

**Figure 9:**
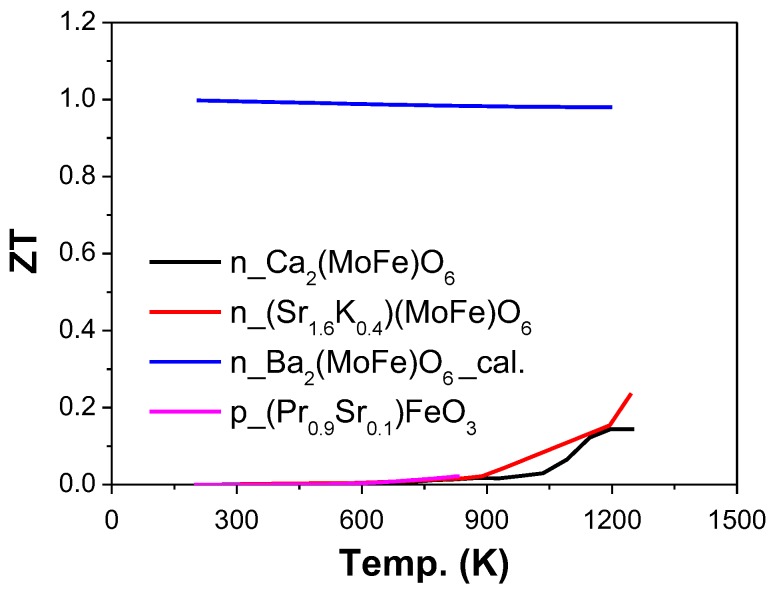
ZT value of AFeO_3_ based oxide perovskite materials, the majority carrier types (n or p) were marked in the legend. The symbol “cal.” means the results come from the calculation.

The reported ZT when Fe was used as B-site was presented in Figure 9. LaFeO_3_ was investigated for TE application. To improve the electrical conductivity, Sr [134,136] and Pr [135] were used as A-site dopant or substitution. The electrical conductivity was increased from 21 to 158 S·cm^−1^ for La_1−x_Sr_x_FeO_3_, when x changed from 0.05 to 0.25, and a ZT value of 0.076 at 1273 K was achieved when x = 0.05 (La_0.95_Sr_0.05_FeO_3_) with S of 228 μV·K^−1^, σ of 21 S·cm^−1^, and κ of 1.85 W·m^−1^·K^−1^ [136].

Double perovskite A_2_FeMoO_6_ was also studied for thermoelectric application. For example, Sugahara et al. reported a ZT value of 0.14 at 1250 K for Ca_2_FeMoO_6_ with S of −108 μV·K^−1^, σ of 270 S·cm^−1^, and κ of 3.1 W·m^−1^·K^−1^ [137]. Additionally, they also presented a ZT value of 0.24 at 1250 K for Sr_1.6_K_0.4_FeMoO_6_ with S of 48 μV·K^−1^, σ of 600 S·cm^−1^, and κ of 3.1 W·m^−1^·K^−1^ [138]. Sahnoun et al. investigated electrical and TE properties of Ba_2_FeMoO_6_ by Wien2K calculations and Boltzmann transport theory in the temperature range of 200 to 1100 K [139]. Their results revealed that Ba_2_FeMoO_6_ could achieve a ZT value of 0.98 at 1000 K with enhanced electrical conductivity [139].

#### 2.4.2. Ni as B-Site (ANiO_3_)

The TE properties of LaNi_1−x_Cu_x_O_3−δ_ (0.2 < x < 0.5) was investigated. Electrical conductivity of 790 S·cm^−1^ was obtained at x = 0.3. Additionally, Cu doping into Ni sites in LaNi_1−x_CuxO_3−δ_ solid solutions can suppress the formation of intermediate secondary phases (the deoxidized La_4_Ni_3_O_10_ and La_3_Ni_2_O_7_ phases) using donor doping effects. Since the increase in the charge valence of a metal ion can be counterbalanced by an equivalent decrease in the formation of oxygen vacancies, Cu doping into Ni ion sites can prohibit the formation of oxygen vacancies. A power factor of 40 µW·K^−2^·m^−1^ was achieved for LaNi_0.8_Cu_0.2_O_3_ at 600 K [140].

#### 2.4.3. Sn as B-Site (ASnO_3_)

**Figure 10:**
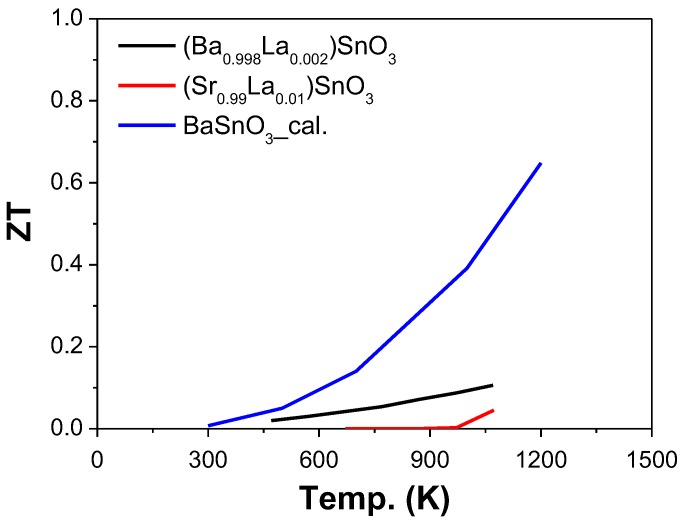
ZT value of ASnO_3_ based oxide perovskite materials, all the materials are n-type semiconductors. The symbol “cal.” means the results come from the calculation.

The reported ZT when Sn was used as B-site was presented in Figure 10. So far, there are several studies that have reported the A-site chemical substitution (e.g., Ba [143] and Sr [144]) to enhance σ. For examples, La was used as A-site dopant in Ba_1−x_La_x_SnO_3_ with x = 0.002, 0.005, 0.008, and 0.010 [143]. The electrical conductivity increased from 120 to 350 S·cm^−1^, when x changed from 0.002 to 0.010. A ZT value reached 0.1 at 1073 K for Ba_0.998_La_0.002_SnO_3_ [143]. The electrical conductivity of Sr_1−x_La_x_SnO_3_ increased from 4 to 120 S·cm^−1^, when x changed from 0.01 to 0.03 [144]. A ZT value of 0.05 was achieved at 1073 K for Sr_0.99_La_0.01_SnO_3_ [144].

Additionally, the thermoelectric transport properties of BaSnO_3_ were calculated using density functional theory combined with Boltzmann transport theory, where the electrical conductivity of BaSnO_3_ is improved dramatically after doping, owing to small effective mass and high mobility [145]. The power factor can reach 1.5 × 10^−3^ W·m^−1^·K^−2^ at 1200 K with a carrier concentration of 1.6 × 10^19^ cm^−3^, giving rise to a ZT value of 0.65 at 1200 K by calculation [145].

#### 2.4.4. Pb as B-Site (APbO_3_)

**Figure 11:**
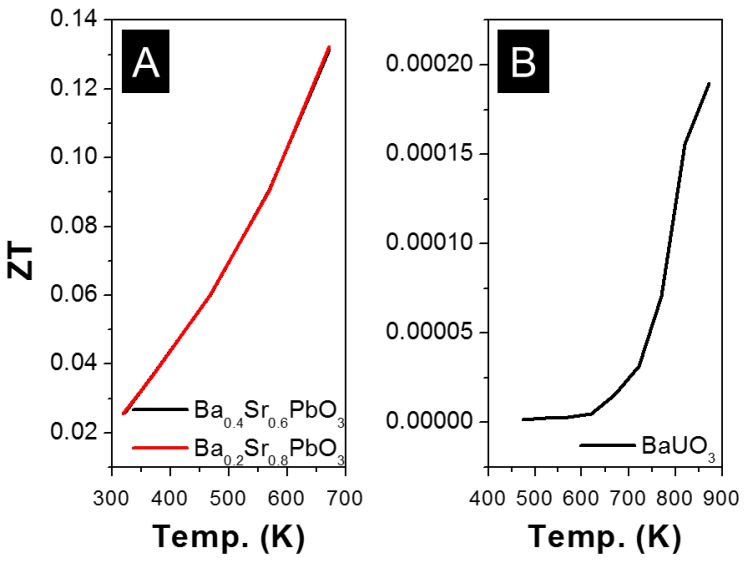
ZT value of (**A**) APbO_3_ and (**B**) AUO_3_ based oxide perovskite materials, all the materials are n-type semiconductors.

The ZT values as a function of temperature for oxide perovskite materials, when Pb was used as B-site, were showed in Figure 11A. Ba has been used as A-site dopant for Sr_1−x_Ba_x_PbO_3_ (0 < x < 1). The electrical conductivity increased from 20 to 1585 S·cm^−1^, when x changed from 0 to 1, a ZT of 0.13 was achieved at 673 K when x = 0.4 (Sr_0.6_Ba_0.4_PbO_3_) [146,147]. Additionally, Bi was used as B-site dopant for Sr_0.7_Ba_0.3_Pb_1−x_Bi_x_O_3_ with 0.00 ≤ x ≤ 0.25, where the electrical conductivity increased from 250 to 400 S·cm^−1^, when x changed from 0 to 0.05 [148].

#### 2.4.5. Bi as B-Site (ABiO_3_)

The TE properties of p-type BaBi_1−x_Sb_x_O_3_ (0.0 ≤ x ≤ 0.5) was investigated at temperatures between 423 K and 973 K. The electrical conduction mechanism is explained by hopping of small polaronic holes localized on the pentavalent cations, and substitution of Bi with Sb would decrease the electrical conductivity but improve the Seebeck coefficient. The electrical conduction in the BaBiO_3_ is due to hopping of small polaronic 6s holes from Bi^5+^ (6s^0^) to Bi^3+^ (6s^2^) with the aid of electron–phonon coupling. Substitution of Bi^5+^ with Sb^5+^ causes the 6s holes to decrease, leading to smaller electrical conductivity and a larger Seebeck coefficient as the Sb content increases. As a result, Sb doping does not enhance the TE properties of BaBiO_3_, a power factor of 30 μW·m^−1^·K^−2^ was obtained for Sb-undoped BaBiO_3_ at 773 K [150].

#### 2.4.6. Mo as B-Site (AMoO_3_)

Kurosaki et al. prepared a polycrystalline sample of perovskite-type barium molybdate (BaMoO_3_), and a ZT value of 0.015 was obtained at 470 K with S of ~−30 μV·K^−1^ [151]. Oba et al. investigated thermoelectric properties of V, Sr, and Mn substituted Sr_2_FeMoO_6_ systems. For Sr_1.4_Ba_0.6_Fe_1−z_Mn_z_Mo_0.8_V_0.2_O_6_ samples, the Seebeck coefficient increased with increasing Mn substitution. The power factor reached 83.2 µW·m^−1^·K^−2^ in the composition of Sr_1.4_Ba_0.6_Fe_0.8_Mn_0.2_Mo_0.8_V_0.2_O_6_ at 973 K [152]. 

#### 2.4.7. Ru as B-Site (ARuO_3_)

Several studies reported the TE properties of ARuO_3_ based materials. For example, polycrystalline SrRuO_3_ was studied, and a ZT value of 0.03 was obtained at 670 K with S of 36 µV·K^−1^ and κ of 5.3 W·m^−1^·K^−1^ [153]. Sr_2_RuYO_6_ and Sr_2_RuErO_6_ were reported to have a S of −475 and −400 µV·K^−1^ at room temperature, respectively [154]. Additionally, La was used as A-site dopant for (Sr_1−x_La_x_)_2_ErRuO_6_ (0 < x < 0.3). The carrier concentration was enhanced from 9.7 × 10^20^ to 2.2 × 10^21^ cm^−3^, when x changed from 0.1 to 0.3, resulting in the electrical conductivity increased from 0.1 to 1.7 S·cm^−1^ at 800 K. A ZT value of ~0.001 was obtained with S of −160 µV·K^−1^ and κ of 7 mW·cm^−1^·K^−1^ for (Sr_0.9_La_0.1_)_2_ErRuO_6_ at 800 K [155].

#### 2.4.8. U as B-Site (AUO_3_)

The TE properties of BaUO_3_ in the temperature range from room temperature to 1000 K was investigated. A ZT value of 0.0002 was obtained at 880 K (Figure 11B) with S of −270 µV·K^−1^, σ of 0.1 S·cm^−1^ and κ of 0.9 W·m^−1^·K^−1^. The low κ was attributed to its phonon glass property [158].

## 3. Hybrid Perovskites

**Figure 12:**
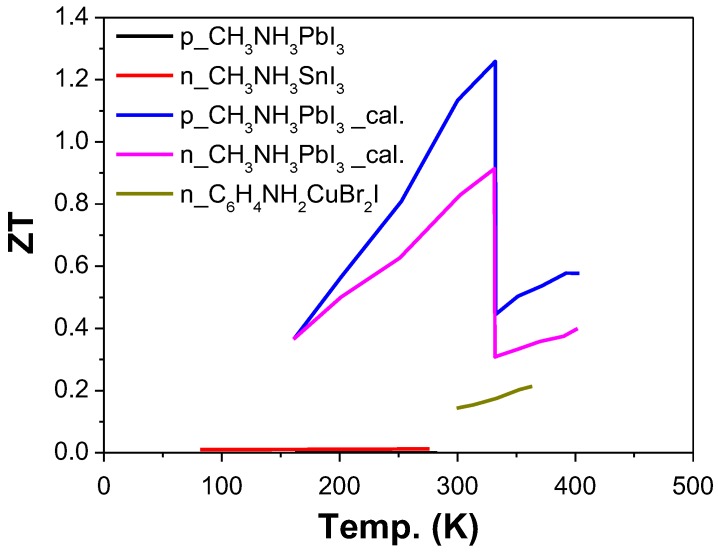
ZT value of hybrid perovskite materials. The majority carrier types (n or p) were marked in the legend. The symbol “cal.” means the results come from the calculation.

The ZT values of hybrid perovskite materials reported by different groups were summarized in Figure 12. Hybrid perovskite materials have a relatively high Seebeck coefficient (700 μV·K^−1^ for CH_3_NH_3_PbI_3_ at 295 K) [160] and low thermal conductivity (0.5 W·m^−1^·K^−1^ for CH_3_NH_3_PbI_3_ at 295 K) [160], but the electrical conductivity is relatively low compared to traditional thermoelectric materials. Studies so far have all focused on how to improve the electrical conductivity through photo-induced or chemical doping strategies. Several hybrid perovskite materials—such as ABI_3_ (A = CH_3_NH_3_ (MA), NH_2_CHNH_2_ (FA) and B = Sn, Pb) [33,34,160,161], CsMI_3_ [162], and C_6_H_4_NH_2_CuBr_2_I [35]—were studied theoretically or experimentally for TE application.

**Figure 13:**
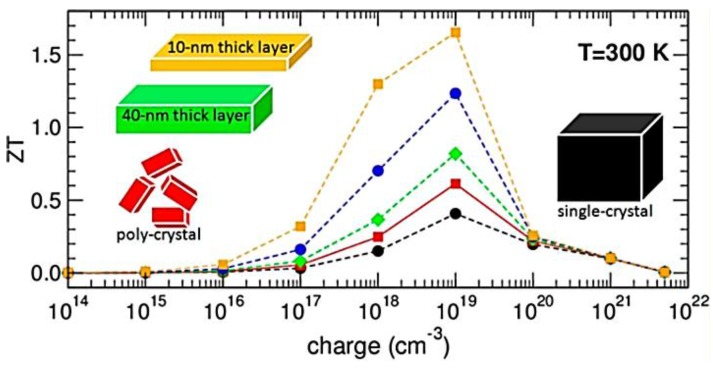
ZT vs. charge concentration for electron-doped CH_3_NH_3_PbI_3_, calculated at 300 K. Black dots and red squares correspond to single crystal and polycrystal CH_3_NH_3_PbI_3_, respectively; the other symbols are for mimicking ZT value in 2D wells of thickness L = 40 nm (green diamonds), 20 nm (blue circles), and 10 nm (orange squares). Reproduced with permission [33]. Copyright 2016, American Chemical Society.

The theoretical analysis showed that the ZT value of hybrid perovskite materials could achieve 0.9 for n-type [33,34,161,162] and 1.25 for p-type [161,162] through chemical doping strategies. For example, the thermoelectric behavior of CH_3_NH_3_PbI_3_ for a wide range of temperatures and carrier concentrations was theoretically analyzed. The results showed optimal carrier concentration of about 10^19^ cm^−3^, leading to an electrical conductivity of 160 S·cm^−1^ and a calculated Seebeck coefficient of −238 μV·K^−1^. In combination with a thermal conductivity ~0.3–0.5 W·m^−1^·K^−1^, this delivers ZT of 0.3–0.9 (Figure 13) [33]. Additionally, the TE properties for CH_3_NH_3_PbI_3_ were theoretically investigated as a function of carrier concentration based on first-principles calculations. The results showed that ZT values of 0.9 and 1.25 could be achieved for n-type and p-type tetragonal CH_3_NH_3_PbI_3_, respectively, with a carrier concentration of 10^19^ cm^−3^ at 330 K [161]. The effect of doping on TE properties of organic–inorganic perovskite iodides ABI_3_ (A = CH_3_NH_3_ (MA), NH_2_CHNH_2_ (FA); B = Sn, Pb) at 300 K were analyzed through calculation. The results indicated that ZT value of 0.44, 0.45, 0.42, and 0.35 can be achieved for n-type (MA)PbI_3_, (MA)SnI_3_, (FA)PbI_3_, and (FA)SnI_3_ at carrier concentrations of 2.3 × 10^19^, 3.3 × 10^19^, 1.2 × 10^19^, and 5.0 × 10^19^ cm^−3^, respectively [34]. Moreover, TE properties of halide perovskite CsMI_3_ (M = Sn and Pb) was investigated by a combination of first-principles calculations and semiclassical Boltzmann transport theory by taking into account both the electron and phonon transport. The calculation showed that the ZT values are up to 0.63 and 0.64 for n-type CsSnI_3_ and CsPbI_3_ at 1000 K with the carrier concentration of 4.2 × 10^19^ and 0.53 × 10^19^ cm^−3^, respectively [162].

Experimentally, the electrical conductivity of hybrid perovskite material was improved by photo or chemical induced doping [35,160].

**Figure 14:**
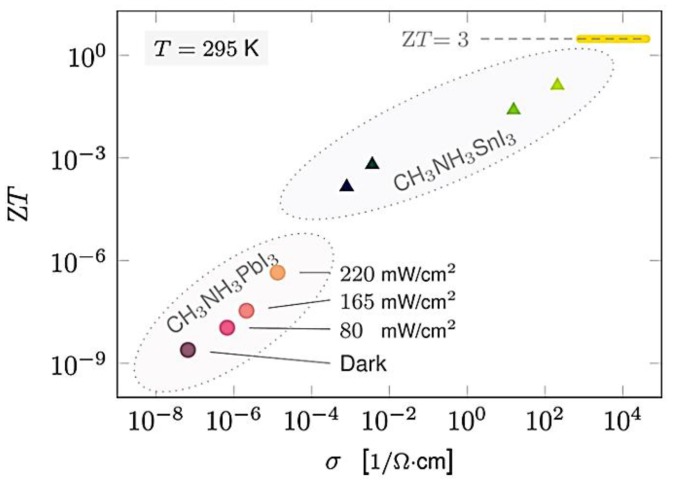
ZT of CH_3_NH_3_PbI_3_ and CH_3_NH_3_SnI_3_ at 295 K, with doping, monitored through the conductivity increase with photo and impurity doping. Red, orange, and yellow correspond to light intensities of 80, 165, and 220 mW·cm^−2^. The dashed line marks ZT = 3. Adapted with permission [160]. Copyright 2015, American Chemical Society.

**Figure 15:**
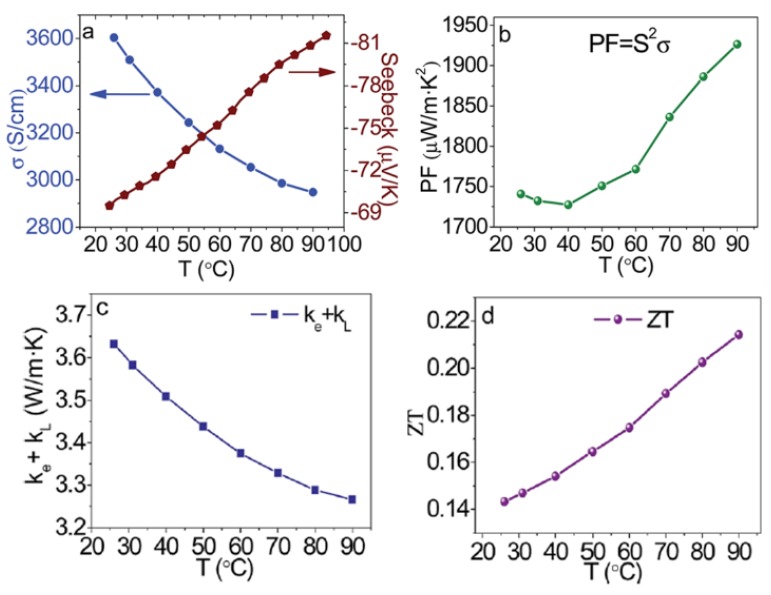
Electrical conductivity, Seebeck coefficient, power factor, calculated thermal conductivity, and ZT at different temperatures for the C_6_H_4_NH_2_CuBr_2_I. Reproduced with permission [35]. Copyright 2017, Royal Society of Chemistry.

The effect of light (photoelectron) and impurity doping on thermoelectric properties of CH_3_NH_3_MI_3_ (M = Pb, Sn) samples were investigated. The electrical conductivity of CH_3_NH_3_PbI_3_ increased from 10^−7^ to 10^−5^ S·cm^−1^ with a carrier concentration of about 10^14^ cm^−3^ from light doping, when changed from dark to light intensities of 220 mW·cm^−2^. The S at room temperature (295 K) decreased upon illumination, from S_dark_ = 820 μV·K^−1^ to S_light_ = 540 μV·K^−1^. Additionally, the electrical conductivity of 10^−3^ S·cm^−1^ was achieved for CH_3_NH_3_SnI_3_ through impurity doping. The largest ZT values obtained was 0.008 at 295 K for CH_3_NH_3_SnI_3_ (Figure 14) [160]. Additionally, the TE properties of n-type organic/inorganic hybrid C_6_H_4_NH_2_CuBr_2_I were investigated. The materials achieved a carrier concentration of 8.7 × 10^20^ cm^−3^. This high charge carrier concentration could be attributed to the self-doping effect caused by the reduction of Cu^2+^ in the C_6_H_4_NH_2_CuBr_2_I film. A part of the Cu^2+^ sites in the film could be replaced by Cu^+^, which can be reduced during the annealing process and affected by the presence of Br^−^. When a part of the Cu^2+^ sites was replaced by Cu^+^, the negatively charged $\;\mathrm{Cu}{\left(+\right)}^{\prime }{}_{\mathrm{Cu}\left(2+\right)}$ defects would be formed in C_6_H_4_NH_2_CuBr_2_I. Furthermore, the former composition becomes C_6_H_4_NH_2_Cu_1−x_^2+^Cu_x_^+^X_3−x_ (X = I^−^ or Br^−^), resulting in vacancies of X^−^ (${V^{\prime }}_{X}$). The formation of negatively charged defects, such as $\;\mathrm{Cu}{\left(+\right)}^{\prime }{}_{\mathrm{Cu}\left(2+\right)}$ and ${V^{\prime }}_{X}$, induce electron doping. The film has an electrical conductivity of ~3.6 × 10^3^ S·cm^−1^ and a Seebeck coefficient of ~−70 µV·K^−1^ at room temperature, resulting in a power factor of 1740 µW·m^−1^·K^−2^. The details about the TE properties were shown in Figure 15. The highest estimated ZT value using calculated thermal conductivity was 0.21 at 363 K [35].

**Figure 16:**
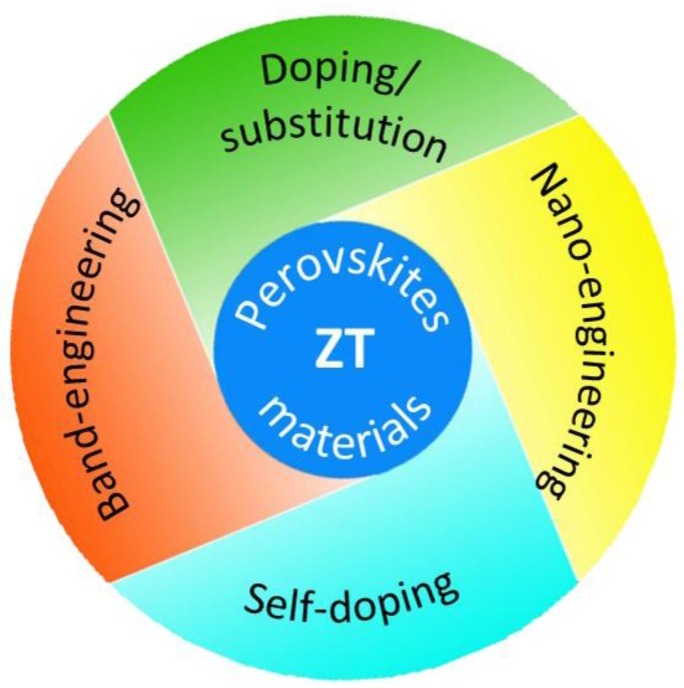
Possible strategies to enhance the thermoelectric ZT value of perovskite materials.

In short, chemical doping is more effective than light doping strategy in hybrid perovskite materials [35,160]. The investigation of hybrid perovskite materials for TE application is just started, but it shows great potential with a power factor reaching 1740 µW·m^−1^·K^−2^ by self-doping strategies [35]. Hybrid perovskites can be promising TE materials with further modification by strategies, such as band-engineering, nano-engineering, doping/substitution, and self-doping (Figure 16).

## 4. Conclusions and Outlook

**Figure 17:**
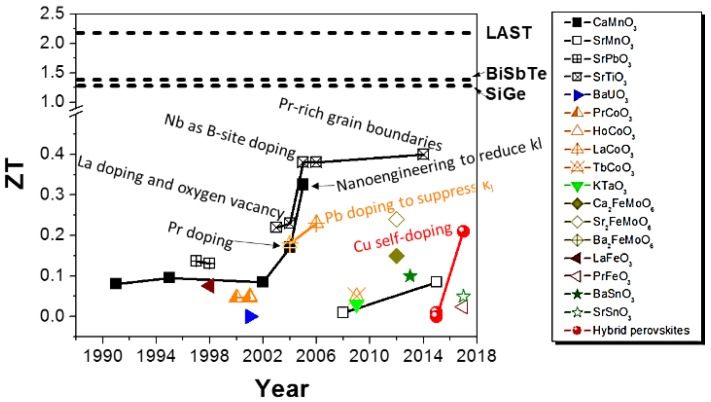
Summary of thermoelectric ZT values of oxide and hybrid perovskite materials by year. The three dashed lines are the ZT value of BiSbTe [163], AgPb_18_SbTe_20_ (LAST) [171], and SiGe [172], which have a different optimum working temperature. The thermoelectric ZT values of all materials were extracted from literature at its optimum working temperature.

The ZT values of the oxide and hybrid perovskite materials from reported experimental data were summarized in Figure 17. The ZT in Figure 17 was read from the literature at its optimum working temperature. Compared to the ZT value of the conventional thermoelectric materials (e.g., BiSbTe [163], AgPb_18_SbTe_20_ (LAST) [171], and SiGe [172]), the ZT values of perovskite materials still need to be further improved.

Oxide perovskites were applied as TE materials in a wide range of temperature (from 100 K–1400 K). The highest ZT value of oxide perovskite materials obtained experimentally so far is ~0.4. The reason for the low ZT value is that oxide perovskite materials have low electrical conductivity and high thermal conductivity. Doping is usually used as a strategy to improve the TE properties, which would normally increase the carrier concentration of materials, thus enhancing the electrical conductivity. In general, A-site doping (e.g., La doping in SrTiO_3_) [40,60,61,62,63,64,65,66,67] is more effective than B-site doping [65,68,69,76] in enhancing the electrical conductivity through increasing the carrier concentration, in some cases when lattice thermal conductivity is dominant, A-site doping can also reduce the thermal conductivity [62,73]. On the other hand, B-site doping [83,107] and grain boundary [59] are typically more effective in introducing phonon scattering, thus suppressing the lattice thermal conductivity (e.g., Pb substitution of La in LaCoO_3_) [83,94,95,102,107,126]. Additionally, the B-site doping can improve the |S| because of increase the effective mass (e.g., Nb substitution of Ti in SrTiO_3_) [65,68]. Moreover, quantum confinement effect can also enhance |S| of perovskite materials dramatically [75]. Furthermore, a small amount of oxygen vacancy in the crystal can have a positive effect on electrical conductivity [38,57,58,59], enhance the Seebeck coefficient [62,63,64], or even suppress the thermal conductivity [79], but it needs to cooperate with other strategies (e.g., A-site or B-site doping) to have a dramatic effect on TE properties. Also, the Pr doping in STO can create Pr rich grain boundaries, which enhances the mobility of the charge carrier significantly [40].

Hybrid perovskite materials started to draw attention for TE application in recent years. It can be a promising TE because of high Seebeck coefficient (700 μV·K^−1^ for CH_3_NH_3_PbI_3_ at 295 K) [160] and low thermal conductivity (0.5 W m^−1^·K^−1^ for CH_3_NH_3_PbI_3_ at 295 K) [160]. To improve the electrical conductivity, photo-induced and/or chemical-induced doping was applied. Chemical doping has experimentally proven to be more effective than light doping strategy [35,160]. For instance, in the film of C_6_H_4_NH_2_CuBr_2_I, a part of the Cu^2+^ sites could be replaced by Cu^+^, then the negatively charged Cu(+)’_Cu(2+)_ defects would form in C_6_H_4_NH_2_CuBr_2_I, resulting in a carrier concentration of ~8.7 × 10^20^ cm^−3^ [35]. The TE properties of hybrid perovskite materials were simulated at high temperature (T > 600 K) [33], but they can be decomposed at about 373 K based on their stability limitations [173]. According to the self-doping mechanism, Pb can be replaced by elements that have multiple valence states, which is a potential choice for self-doping elements in hybrid perovskites. Other strategies to improve TE properties of materials, such as band-engineering and nanoengineering, can be applied to hybrid perovskite materials (Figure 16). The theoretical calculation results claimed that hybrid perovskite materials could achieve a ZT value of 0.9 for n-type [33,34,161,162] and 1.25 for p-type [161,162]. Hybrid perovskite materials, which have low capital cost and can be quickly processed by energy cost methods, can be a promising candidate for TE application near room temperature range. Hybrid perovskite materials have the potential for flexible/wearable thermoelectric generator/cooling devices.

## Figures and Tables

**Table 1:** Literature data about TE properties of perovskite material.

Ref.	Materials	Seebeck (µV·K^−1^)	Electrical Conductivity (S·cm^−1^)	Thermal Conductivity (W·m^−1^·K^−1^)	Power Factor (µW·K^−2^·m^−1^)	ZT	Measure Temp. (K)
[38]	La-Doped SrTiO_3_	−260					298
[57]	Sr_0.85_La_0.15_TiO_3_	175	400	3		0.28	873
[58]	SrTiO_3_	210	250		200		300
[59]	reduced graphene oxide—SrTiO_3_	−380	30			0.09	760
[40]	Sr_0.875_Pr_0.125_TiO_3_	−80	3700			0.4	323
[60]	La-doped SrTiO_3_	980			0.39		298
[61]	Sr_0.9_La_0.1_TiO_3_	−250					1000
[62]	Sr_0.9_Dy_0.1_TiO_3_ R = (La, Sm, Gd, Dy, Y)	−160	500	2.7		0.22	573
[63]	Sr_0.95_La_0.05_TiO_3_	250	150	4.2	800	0.15	780
[64]	Sr_0.9_La_0.1_TiO_3_	−225	300	3.2		0.21	750
[65]	La-doped SrTiO_3_	−300	80	3.1		0.27	1073
[66]	Sr_0.96_Ce_0.04_TiO_3_	−65					300
	Sr_0.98_La_0.02_TiO_3_	−138					300
[67]	Sr_2_CoTiO_6_	60	10				1200
[68]	Sr(Ti_0.8_Nb_0.2_)O_3_	−200		3.5	1300	0.37	1000
[69]	BaNb_0.01_Ti_0.99_O_3_	−640					290
[70]	BaTiO_3_	−550					300
[71]	Sr_0.9_Y_0.1_TiO_3_	−130			120		420
[72]	Ba_0.3_Sr_0.6_La_0.1_TiO_3_	−110		4.4		0.13	420
[73]	Sr_0.45_Ca_0.45_La_0.1_TiO_3_	−195	250	3.7		0.22	850
[74]	Sr_0.98_La_0.02_TiO_3_	−260	500	11		0.09	298
[75]	SrTiO_3_/SrTi_0.8_Nb_0.2_O_3_/SrTiO_3_	−850	1400	12		2.4	300
[76]	Sr(Ti_0.8_Nb_0.2_)O_3_			3		0.35	1000
[77]	Sr_0.2_Eu_0.8_Ti_0.8_Nb_0.2_O_3_	170	0.5				1000
[78]	SrTi_0.9_Ta_0.1_O_3_	−175	300	4.4		0.17	752
[79]	BaTiO_3_	−620	0.17				300
[80]	PbTiO_3_	110					300
	BaTiO_3_	110					300
	SrTiO_3_	100					300
[81]	SrTiO_3_	−500					300
[82]	Nb-doped SrTiO_3_	−540					1200
[83]	Sr_0.95_La_0.05_Ti_0.98_Mn_0.02_O_3_	−150	833	3.9	20	0.15	300
[84]	La_0.25_Sr_0.75_TiO_3_	450					300
[85]	n-type SrTiO_3_	300				0.7	1400
[86]	Sr_2_TiFeO_6_	280	1.2		10		1130
	Sr_2_TiCoO_6_	60	20		5.4		750
[39]	Ba_0.25_Sr_1.75_FeTiO_6_	800			15		1123
[87]	Sr_2_TiMoO_6_	−10.8	960		11		1223
[88]	SrTiO_3_	−150					1000
[89]	SrTi_0.5_Co_0.5_O_3_	200				1.36	800
[90]	(Tb_0.1_Ca_0.9_)MnO_3_	−140	−0.18			0.13	950
	(Ho_0.1_Ca_0.9_)MnO_3_	−110	−0.18			0.08	950
	(Y_0.1_Ca_0.9_)MnO_3_	−130	−0.2			0.15	950
[91]	(Ca_0.9_Bi_0.1_)MnO_3_	80	10	4		0.095	1173
[92]	Sr_0.9_Ce_0.1_MnO_3_	180	100				1273
[93]	CaMnO_3_	−520					667
[94]	Ca_0.85_Pr_0.15_MnO_2.98_	−130	111	1.5		0.17	1100
[95]	Ca_0.9_Yb_0.1_MnO_3_	−150	133	1.6		0.16	970
[96]	Ca_0.8_Dy_0.2_MnO_3_	−135	270		510		1073
[97]	Pr_0.3_Sr_0.7_MnO_3_	−75	250	1.6		0.085	1073
[98]	Sm_0.25_Ca_0.75_MnO_3_	−380					65
[99]	CaMn_0.88_Mo_0.12_O_3_	−240	0.011				100
[100]	YNi_0.3_Mn_0.7_O_3_	90					357
[101]	SrMnO_3_	−110	50				300
[102]	CaMn_0.85_Mo_0.15_O_3_ (Sm_0.2_Ca_0.8_MnO_3_, Pr_0.15_Sr_0.85_MnO_3_)	−270	10^−4^	0.6			80
[103]	Ca_0.96_Bi_0.04_MnO_3_	−170	66.7	3.6	300	0.086	1000
[104]	CaMn_0.96_Mo_0.04_O_3_	−90		3.4		0.012	270
	CaMn_0.94_Ru_0.06_O_3_	−140		5.4		0.0085	330
[105]	CaMn_0.98_Ta_0.02_O_3_	−190	29			0.05	1000
	CaMn_0.98_Nb_0.02_O_3_	−205					1000
[106]	Ca_0.4_Sr_0.6_Mn_0.96_Mo_0.04_O_3_	−110	50				220
[107]	CaMn_0.98_Nb_0.02_O_3_	−255	31			0.32	1050
[108]	SrMn_0.7_Ru_0.3_O_3_	−40	50	1.8		0.01	370
[109]	Ca_0.9_Nd_0.1_MnO_3_	−150	160		398		1123
[110]	Sr(Mn_0.975_Mo_0.025_)O_3_	−120	0.13	5		0.003	400
[111]	Ca_0.8_Nd_0.2_MnO_3_	−62	280	1.3		0.17	873
[112]	Sr_0.9_Ti_0.1_MnO_3_	−340	2.25		25		800
[113]	(Pr_0.9_Ca_0.1_)CoO_3_	106	220	1.9		0.047	358
[114]	Pr_0.9_Ca_0.1_CoO_3_ [RCoO_3_ (R = Pr, Nd, Sm, Gd, Dy, Ho)]	125	220	1.6		0.046	450
[115]	TbCoO_3_ DyCoO_3_	80	200	1.6		0.05	873
[116]	Nd_0.95_Sr_0.05_CoO_3_	130					400
[117]	Nd_0.99_Sr_0.01_CoO_3_	300					290
	Pr_0.99_Sr_0.01_CoO_3_	450					270
[118]	LaCoO_3_	1200					100
[119]	La_0.975_Sr_0.025_CoO_3_	280					300
[120]	La_0.95_Sr_0.05_CoO_3_	720	100				280
[121]	Ho_0.9_Ca_0.1_CoO_3_	220	20	0.75		0.051	573
[122]	La_0.875_Sr_0.125_CoO_3_	100		6		0.035	230
[123]	La_0.95_Sr_0.05_CoO_3_	720	20	0.037		0.18	300
[124]	La_0.95_Sr_0.05_CoO_3_	170		1.2		0.033	300
[125]	La_0.9_Sr_0.1_CoO_3_	120		1.5		0.046	300
[126]	La_0.9_Pb_0.1_CoO_3_	110	333	0.8		0.23	575
[127]	Nd_0.995_Ca_0.005_CoO_3_	500	1.1				300
	LaCo_0.99_Mn_0.01_O_3_	−200					300
[128]	La_0.97_Ba_0.03_CoO_3_	80	40		80	0.08	420
[129]	YCo_0.98_Ni_0.02_O_3_	900					300
[130]	LaCoO_3_	600	0.067				300
	La_0.98_Sr_0.02_CoO_3_	350	0.3				300
	La_0.99_Ce_0.01_CoO_3_	−300	0.03				300
	LaCo_0.995_Ga_0.005_O_3_	480					300
	LaCo_0.995_Ti_0.005_O_3_	−200					300
[131]	LaCo_0.92_Ni_0.08_O_2.9_	220	33.3	0.35		0.2	300
[132]	La_0.94_Sr_0.06_CoO_3_	180		2		0.048	300
[133]	La_0.9_Sr_0.1_CoO_3_					0.046	300
[134]	La_0.9_Sr_0.1_FeO_3_	380					1173
[135]	Pr_0.9_Sr_0.1_FeO_3_	140		0.8		0.024	850
[136]	La_0.95_Sr_0.05_FeO_3_	230		1.8		0.076	1273
[137]	Ca_2_FeMoO_6_	−108	300	3.2		0.14	1250
	Ca_1.9_Sr_0.1_FeMoO_6_	−110	250	3.0		0.14	1250
	Ca_1.8_Sr_0.2_FeMoO_6_	−100	260	2.8		0.14	1250
[138]	Sr_1.6_K_0.4_FeMoO_6_	−48		3.1	450	0.24	1250
[139]	Ba_2_FeMoO_6_	−1350				0.995	300
[140]	LaNi0.8Cu0.2O_3_	−26	600		40		600
[141]	BaSn_0.99_Co_0.01_O_3_	1000					700
[142]	Sr_0.98_La_0.02_SnO_3_	−80	0.03				300
[143]	Ba_0.998_La_0.002_SnO_3_	−170	150	4		0.1	1073
[144]	Sr_0.99_La_0.01_SnO_3_	−80	1.5	3.6	120	0.05	1073
[145]	BaSnO_3_	−130	300	3.4	1400	0.65	1200
[146]	Ba_0.4_Sr_0.6_PbO_3_	125	250	2		0.13	673
[147]	Ba_0.2_Sr_0.8_PbO_3_	−190	79	1.8		0.13	680
[148]	Sr_0.7_Ba_0.3_Pb_0.99_Bi_0.01_O_3_	−70			390		900
[149]	Ba_0.77_K_0.23_BiO_3_	3.2	10				290
[150]	BaBi_0.9_Sb_0.1_O_3_	260			10		850
[151]	BaMoO_3_	−30				0.015	1000
[152]	Sr_1.4_Ba_0.6_Fe_0.8_Mo_0.8_Mn_0.2_V_0.2_O_6_	−58	316		83.2		973
[153]	SrRuO_3_	36		5.3		0.03	1200
[154]	Sr_2_RuYO_6_	−250					1200
	Sr_2_RuErO_6_	−250					1200
[155]	(Sr_0.95_La_0.05_)_2_RuErO_6_	−160				0.001	800
[156]	AgNbO_3_	700 −500					800
	AgTaO_3_	710					800
[157]	K_0.991_Ba_0.009_TaO_3_	200	333			0.03	300
[88]	KTaO_3_	−280					1000
[158]	BaUO_3_	−170	0.1	0.8		0.0002	880
[159]	BaCe_0.95_Y_0.05_O_3_	−220					873
[35]	C_6_H_4_NH_2_CuBr_2_I	−82	2950	3.25		0.21	363
[33]	CH_3_NH_3_PbI_3_-n-type	−238	160	0.1	8.4	0.61	300
	p-type	181	94	0.06	3.1	0.25	300
	n-type	−362	89	0.145	10.5	1.61	600
	p-type	295	41	0.06	3.6	0.71	600
	n-type	−428	68	0.151	11.3	2.56	800
	p-type	358	25	0.04	3.3	1.08	800
[160]	CH_3_NH_3_SnI_3_	720	0.001	0.08		0.01	295
	CH_3_NH_3_PbI_3_	700	10^−7^	0.5		10^−7^	295
[34]	(MA)PbI_3_	200				0.44	298
	(MA)SnI_3_	200				0.44	298
	(FA)PbI_3_	100				0.43	298
	(FA)SnI_3_	150				0.35	298
[161]	CH_3_NH_3_PbI_3_-n-type	80	1.2			0.9	330
	CH_3_NH_3_PbI_3_-p-type	50	1			1.25	330
[162]	CsSnI_3_			0.18		0.63	1000
	CsPbI_3_			0.1		0.64	1000
